# Oral Health-Related Quality of Life from Young Adulthood to Mid-Life

**DOI:** 10.3390/healthcare11040515

**Published:** 2023-02-09

**Authors:** Chuen Lin Hong, W. Murray Thomson, Jonathan M. Broadbent

**Affiliations:** Department of Oral Sciences, Faculty of Dentistry, University of Otago, Dunedin 9016, New Zealand

**Keywords:** quality of life, cohort studies, inequalities, epidemiology, social determinants

## Abstract

Quality of life varies with time, often worsening, and is affected by circumstances, events, and exposures at different stages of life. Little is known about how oral health-related quality of life (OHRQoL) changes during middle age. We investigated OHRQoL changes from age 32 to 45 years among participants in a population-based birth cohort, along with clinical and socio-behavioural associations. Generalised estimating equation models were used to investigate the association between OHRQoL (assessed at ages 32, 38, and 45 years; n = 844), and the socioeconomic position in childhood (up to age 15 years) and adulthood (ages 26 through to 45 years), dental self-care (dental services utilisation and tooth brushing), oral conditions (such as tooth loss), and experiencing a dry mouth. The multivariable analyses were controlled for sex and personality traits. At each stage of life, those of a lower socioeconomic status were at greater risk of experiencing OHRQoL impacts. Those who engaged in favourable dental self-care habits (the regular use of dental services and at least twice daily tooth brushing) experienced fewer impacts. A social disadvantage at any stage of life has enduring deleterious effects on one’s quality of life in middle age. Ensuring access to timely and appropriate dental health services in adulthood may reduce the impacts of oral conditions on one’s quality of life.

## 1. Introduction

Oral conditions have debilitating impacts on people’s day-to-day lives. The impact of the social gradient in individuals’ experiences of their quality of life is well documented, with those who are socially disadvantaged reporting greater impacts [1,2,3,4,5]. These inequalities persist even after controlling for oral disease experience and dental services utilisation [6]. Moreover, people with differing personality characteristics are likely to experience the impacts on their quality of life differently [7], and sex differences are commonly observed [6,8]. 

A range of models have been proposed to explain the association between oral health and quality of life. Chen and Hunter’s conceptual model posited that the association was dependent upon social characteristics (education, occupation, etc.) and oral health behaviours (brushing, flossing, and use of dental services), but did not consider how these factors act across time [9]. The theoretical model of Sischo and Broder [10] also considered these issues, as well as aspects of mental health, and highlighted the need for longitudinal research into oral health-related quality of life (OHRQoL). Further to this, Broadbent et al. [11] attempted to elaborate how a chain of risk acts intergenerationally, with parental occupation and health-related beliefs affecting the oral health behaviours and social characteristics of their children, conferring the risk of oral health conditions and (ultimately) impacting the OHRQoL by the time those children reach adulthood.

Broadbent et al. [11] considered OHRQoL only at one point in adulthood, at age 38. However, OHRQoL may change with a changing oral health status and personal circumstances at different stages of life. People who lose teeth may learn to live with this over time; alternatively, they may find tooth loss to be an issue that causes them greater difficulty as they age and lose further teeth. People’s financial circumstances may change throughout life, affecting their ability to receive timely oral health care services to meet their oral health care needs. 

The life course approach [12] can be useful in understanding how changes in oral conditions affect people’s quality of life over time, but few studies have done so. One example is the Newcastle Thousand Families cohort, which had reported socio-economic status (SES) to be associated with OHRQoL at age 50 years, with apparent differences by sex [8], but little evidence for the effects of social mobility [13]. 

The aim of this study was to describe changes in OHRQoL between age 32 and 45 years and investigate the clinical and socio-behavioural antecedents of these.

## 2. Materials and Methods

The participants were members of the Dunedin Multidisciplinary Health and Development Study (Dunedin Study), a longitudinal study of a birth cohort born in Dunedin, New Zealand. Between 1 April 1972 and 31 March 1973, 1037 (91% of eligible births; 52% male) participated in the first follow-up at age 3 years; these constituted the base sample for the remainder of the study. Cohort families represented the full range of socioeconomic status (SES) in New Zealand’s South Island. Over 90% of cohort members identified as New Zealand European or “white”, while 7.5% self-identified as being Māori. This matches the ethnic distribution of the South Island of New Zealand. Follow-ups were done at ages 5, 7, 9, 11, 13, 15, 18, 21, 26, 32, 38, and 45 years, when we assessed 94% of the surviving 1007 study members. The Otago Research Ethics Committee, Dunedin, New Zealand, granted ethics approval for each assessment phase. The study members provided informed consent before participating [14,15].

The short-form oral health impact profile (OHIP-14) [16] was used to assess OHRQoL at age 32, 38, and 45 years. The OHIP-14 questionnaire has 14 items representing a total of seven domains. Responses were measured on a five-point Likert-type scale (never = 0, hardly ever = 1, occasionally = 2, fairly often = 3, very often = 4). Two summary measures were computed: first, the mean OHIP-14 scores, and second, the prevalence of one or more items reported ‘fairly often’ or ‘very often’. To assess the changes in the two summary measures between ages 32 and 45 years, two OHIP-14 ‘change’ variables were computed. First, using the mean OHIP-14 summary score, the study members were categorised into three OHIP-14 change categories using the minimally important difference of five scale points [17]. Those who had an increase of five scale points were allocated to the ‘improved’ group; those with a decrease of five scale points were in the ‘worsened’ group; and the rest were allocated the ‘stable no change’ group. Second, based on the prevalence of OHIP-14 impacts ‘fairly often’ or ‘very often’ at age 32 and 45 years, the study members were allocated to one of four change groups. Those who reported no OHIP-14 impacts at both ages were designated to the ‘stable no impacts’ group. Those who reported 1+ impacts at age 32 years and no impacts at age 45 years were categorised as the ‘resolved’ group, while those who reported no impacts at age 32 years and incident impact at age 45 years were designated the ‘incident impacts’ group. The ‘stable impacts’ group comprised study members who reported 1+ impacts at both ages. 

Clinical dental data from age 32, 38, and 45 years were used. Teeth were examined for dental caries and restorations by calibrated examiners using the WHOs criteria. At age 45, the intra-examiner reliability in the scoring of the count of DMF tooth surfaces was 0.99 for each of the three examiners. The inter-examiner reliability correlation coefficient scores were 0.97 (examiners 1 and 2), 0.95 (examiners 1 and 3), and 0.99 (examiners 2 and 3). Full-mouth periodontal examinations involving measurements of gingival recession and probing depth at three sites per tooth (excluding third molars) were conducted at age 32, 38, and 45 years. The combined attachment loss (CAL) for each site was calculated by summing gingival recession and probing depth. The extent of ≥5 mm CAL (percent of measured sites with 5+ mm of periodontal attachment loss) was used in this study. 

Childhood SES was measured with a six-point scale which assessed parents’ self-reported occupational status [18]. The variable used was the average of the highest SES level of either parent, assessed repeatedly from birth to age 15 years [19]. SES in adulthood (at ages 26 to 45 years) was determined using the New Zealand Socio-Economic Index 2006 [20], an occupation-based measure. At each assessment, the study members were categorised into one of three SES categories (high, medium, and low). Those who were in the lowest adulthood SES group for less than 50% of the assessments (at which they participated) were assigned to the ‘<50%’ adulthood group, and the rest to the ‘≥50%’ group. 

The dental visiting pattern was determined using self-reported dental visiting behaviours at age 26, 32, 38, and 45 years. The study members were asked whether they usually visited the dentist for a check-up or only for a dental problem, together with the number of months since their last visit. For each of those ages, routine attenders were identified as those who usually visited for a check-up and had made a dental visit during the previous 12 months. Those who were categorised as routine attenders at all assessments (at which they participated) were assigned to the ‘always routine attenders’ group, while the study members who were never routine attenders were designated to the ‘never routine attender’ group. The rest were categorised as the ‘sometimes routine attender’ group. 

Self-reported tooth brushing frequency at age 26, 32, 38, and 45 years was assessed by asking “When do you usually brush your teeth?” (response options: “More than once a day”, “Once a day”, “Not every day”, “Less than once a week”, and “Never”). For the analyses reported here, the participants were categorised as brushing “more than once per day” or “once or less per day”.

At age 32, 38, and 45 years, the study members were asked the question “How often does your mouth feel dry?” (response options “Always”, “Frequently”, “Occasionally”, or “Never”). Those who responded “Always” or “Frequently” were designated as “xerostomic” [21]. 

Study members completed a 177-item modified version (‘Form NZ’) of the multidimensional personality questionnaire (MPQ) at age 26 years [22,23]. Its ten independent subscales define the three superfactors of ‘constraint’, ‘negative emotionality’, and ‘positive emotionality’. The standardised MPQ superfactor scores were used in this study.

The statistical significance of differences in the OHIP-14 score by sociodemographic characteristics (sex, SES in childhood and adulthood) and the use of dental services in adulthood was determined using the Kruskal–Wallis test, as for changes in the OHIP-14 score between the assessments by the use of dental services, SES in adulthood, and oral health. The statistical significance of differences in the prevalence of OHIP-14 impacts (fairly or very often) was tested using the Chi-Square test. The observed OHIP-14 scores over time depend on the person’s characteristics; accordingly, generalised estimating-equation (GEE) models were fitted under the exchangeable correlation matrix. Two separate models were used to quantify the association between the OHIP-14 mean scores (and impact prevalence) and the socio-demographic characteristics (Stage I), and the use of dental services, clinical oral status, tooth brushing, and experiencing a dry mouth (Stage II/final model). The negative binomial distribution was used for the GEE model of the OHIP-14 scores, and the binomial distribution was used for modelling the prevalence of OHIP-14 impacts. Both models were controlled for sex, age, and personality traits (using standardised MPQ superfactor scores for ‘constraint’, ‘negative emotionality’, and ‘positive emotionality’). For reasons of data presentation, the estimates for untreated caries, restorations, tooth loss, and the extent of periodontal attachment loss were all presented as ‘per 10’. All the analyses were conducted using Stata/SE 17 software (StataCorp, College Station TX, USA).

## 3. Results

Of the 938 study members (94%) who took part in the age 45 assessment, 896 (96%) were dentally examined. Complete OHIP-14 data were available for 884 individuals at age 32, 892 at age 38, and 856 at age 45 years. The current analyses were limited to the 844 individuals who were dentally examined at age 45 and had complete OHIP-14 data available from all three ages. Those who were excluded due to incomplete OHIP-14 data did not differ from those included by sex, childhood, and adulthood SES. 

There was a gradient in the OHIP-14 scores by SES (Table 1). At age 32 years, the absolute difference in the mean OHIP-14 scores between the high and low childhood SES categories was 2.8. By age 38 and 45 years, it was 4.7 and 5.1, respectively. For adulthood SES, the absolute difference between the two SES groups was 4.1 at age 32 years and, by age 38 and 45 years, it was 5.4 and 5.6, respectively. Similarly, a SES-related divergence in OHIP-14 impacts was observed. Always-routine users of dental services had significantly lower mean OHIP-14 scores and the prevalence of OHIP-14 impacts than those who were not. 

From age 32 and 45 years, one in five study members were in the *worsened* group while two in five were in the *improved* group (Table 2). The proportion of adulthood spent as a routine attender was significantly associated with the three OHIP-14 score change categories. Those who experienced an improvement in the OHIP-14 score had a 12-fold lower mean change in the number of decayed surfaces than the *worsened* group. The “stable no change” group had four-times more filled teeth than the *worsened* group. The *stable* group had the lowest mean change in the number of teeth lost due to caries, with the mean change score being two times lower than that of the *worsened* group. 

Adulthood SES, the use of dental services, and oral health status were associated with the OHIP-14 impact change categories (Table 3). A higher proportion of study members who had spent less than half of their adulthood in the lowest SES group were in the *stable no impacts* group. None of the always-routine attenders was in the *stable impacts* group, while nearly one in five never-routine attenders were. The *stable impacts* group experienced the lowest mean change in the number of filled surfaces during the 13-year period, but they had greater tooth loss from age 32 to 45 than those in the other groups. Study members in the *stable impacts* group experienced the highest mean change in the extent of sites with 5 + mm CAL. 

The OHIP-14 score was associated with SES, dental caries, dry mouth, and dental self-care habits (Table 4). In the fully adjusted model, the study members in the lowest childhood SES group or who had spent ≥50% of adulthood in the lowest SES group had higher OHIP-14 scores than those who were better off. Those who had never accessed routine dental care throughout adulthood also had greater OHIP-14 scores. 

SES in both childhood (being in the low SES group) and adulthood were associated with greater OHIP-14 impacts (Table 5). The routine use of dental services in adulthood ‘sometimes’ was associated with a lower risk for OHIP-14 impacts. The experience of dental caries, dental restorations, caries-related tooth loss, and dry mouth were associated with a greater risk for OHIP-14 impacts. Toothbrushing at least twice daily was associated with a lower risk for impacts.

## 4. Discussion

This study investigated OHRQoL changes in middle-aged individuals and their antecedents. Almost two-thirds of the study members (63.9%) experienced a change in the burden of OHRQoL of five OHIP-14 points or greater (the minimally important difference, Locker, Jokovic [17]). The utilisation of dental services and tooth brushing were associated with changes in OHRQoL; these are modifiable risk factors that have repeatedly been recognised as targets for intervention. Those who did experience impacts on OHRQoL were more likely to be of low SES. This evidence supports the appropriateness of efforts to improve the access to dental care and the support of good self-care practices among more deprived population groups. 

Before discussing the implications of these findings, it is important to consider the study’s strengths and weaknesses. A strength is the use of a broad range of clinical indicators of oral health and self-reported oral health measures from childhood as well as in adulthood, enabling an investigation of the influences of socio-behavioural factors on changes in OHRQoL during an important part of the life course. Further, this study used a population-based birth cohort with a high participant retention, meaning the findings are likely to be generalizable to similar populations.

A possible criticism of this research might be that a proportion of the observed changes in OHRQoL over time might represent a response shift rather than actual changes in the health state. However, others have reported that longitudinal changes in OHIP-14 scores are likely to indicate actual changes in the oral health state, rather than response shifts [24]. Consistent with this assertion, we observed concomitant changes in the oral disease state with changes in the OHIP-14 scores. Furthermore, we controlled for personality characteristics (specifically positive and negative emotionality) and sex, minimising the likelihood of bias in our findings due to those factors. Another criticism may be that the use of three OHIP-14 score ‘change’ categories in the bivariate analyses may mask some important differences within the categories. For example, within the *stable no change* group, some may have stable low scores which do not change, and others might have stable high scores which do not change. However, the multivariate models accounted for this concern by using the OHIP-14 total score to represent the severity of impacts on OHRQoL across the 13 years. 

Numerous cross-sectional studies have reported poorer OHRQoL among socially disadvantaged adults than those who are better off [1,2,4,25], and our findings were consistent with these. They were also consistent with the few studies to have longitudinally investigated OHRQoL, in that OHRQoL was associated with tooth loss [26] and an unmet treatment need [27]. 

We found that childhood disadvantage was associated with widening inequalities in OHRQoL into middle age. Study members of high childhood SES had lower mean OHIP-14 scores and OHIP-14 impact prevalence in adulthood than their disadvantaged counterparts, suggesting that a socioeconomically compromised start to life may have long-lasting impacts on OHRQoL well into adulthood. This is consistent with the findings from the Newcastle Thousand Families Study [8] which also showed that having a more advantaged socio-economic position in early life (birth and infancy) was associated with better OHRQoL at age 50 years. The findings of a previous Dunedin study showed that being of persistently low SES was associated with poorer oral health in adulthood [28,29]. Those who are better off may also be more likely to have access to dental care or practice better oral health-related self-care behaviours. Sex differences in OHRQoL have also been reported [6,8,30,31]. For these reasons, the current analyses accounted for these influences through multivariate modelling. After controlling for sex, and other possible direct and indirect influences from oral health and oral health-related behaviours, our findings offer support that both early life and proximal socioeconomic circumstances continued to have a bearing on OHRQoL. 

We also observed associations between the routine utilisation of dental services in adulthood and better OHRQoL, which is consistent with previous Dunedin Study reports [32,33,34], as well as the findings from a Swedish cohort study [35]. Those with favourable dental visiting behaviours experience better oral health and, hence, a better quality of life. We accounted for the possible influence of the ‘healthy user effect’ on OHRQoL by controlling for the oral health status and tooth brushing behaviours, a proxy for self-care. Overall, the mean OHIP-14 scores were highest for those who were never routine attenders in adulthood. While perhaps part of the observed differences in OHRQoL between the always and never routine attenders in adulthood may be explained by a healthy user effect, our findings support the notion that the routine access to dental health services in adulthood may act to protectively reduce the burden and impacts of oral conditions. New Zealand has arguably one of the most comprehensive systems of oral health care for children and young people up to 17 years of age, but that ceases thereafter. Mejia and Elani [3] reported wider socio-economic disparities in the experience of dental caries among New Zealand adults than those from Australia, Canada, and the United States, suggesting that a good start to life in terms of access to oral health services is not sufficient to ensure good oral health (and OHRQoL) for life. Our findings support improving the access to public dental services in adulthood, and any such steps to improve accessibility and availability to timely and appropriate dental care are likely to lead to better OHRQoL. It is noteworthy that the study members who experienced worsening OHRQoL during adulthood (the *worsened* OHIP-14 group) also experienced the most tooth loss, untreated caries, and periodontal disease, yet had received the fewest dental restorations.

## 5. Conclusions

The findings provide further evidence that a social disadvantage at any stage of life has deleterious effects on OHRQoL in middle age, that OHRQoL differs between the sexes, and that the utilisation of dental care is an important mediator of the association between poor dental health and impacts on OHRQoL. Improving the access to timely and appropriate dental health services is likely to have benefits for one’s quality of life. In particular, toothbrushing and the use of dental services on a regular basis are associated with a lower risk for impacts on OHRQoL even when controlling for the oral health state and socioeconomic factors, and these represent clear areas where an intervention could improve OHRQoL for those affected by oral conditions or who are at a relative socioeconomic disadvantage. The failure to address social deprivation will only perpetuate and exacerbate the OHRQoL inequality gap.

## Figures and Tables

**Table 1:** Mean oral health impact profile (OHIP-14) scores and impact prevalence at ages 32, 38, and 45 years by participant socio-demographic characteristics.

	Mean OHIP-14 Score (sd)			1+ OHIP-14 Impacts Fairly⁄Very Often (%)		
	32 y	38 y	45 y	32 y	38 y	45 y
Total	7.8 (7.8)	8.1 (8.1)	8.5 (8.5)	196 (23.2)	191 (22.6)	204 (24.2)
Sex						
Male	7.9 (7.6)	7.9 (7.9)	8.6 (8.6)	94 (22.5)	84 (20.1)	103 (24.6)
Female	7.7 (8.1)	8.3 (8.4)	8.4 (8.5)	103 (23.9)	107 (25.1)	101 (23.7)
*P* value	0.738	0.464	0.762	0.617	0.081	0.752
Childhood socioeconomic status						
High	6.4 (7.7)	5.8 (6.4)	6.0 (7.2)	19 (13.3)	18 (12.6)	23 (16.1)
Medium	7.6 (7.3)	7.9 (7.7)	8.4 (8.1)	122 (23.0)	121 (22.8)	117 (22.0)
Low	9.2 (8.8)	10.5 (9.9)	11.1 (9.9)	52 (23.0)	50 (30.3)	62 (37.6)
*P* value	<0.001	<0.001	0.004	<0.001	<0.001	<0.001
Proportion of adulthood in low socioeconomic group						
<50%	7.0 (7.3)	7.0 (7.4)	7.4 (7.8)	126 (18.5)	129 (19.0)	134 (19.7)
≥50%	11.1 (8.9)	12.4 (9.5)	13.0 (10.0)	70 (42.7)	62 (37.8)	70 (42.7)
*P* value	<0.001	<0.001	<0.001	<0.001	<0.001	<0.001
Proportion of adulthood as routine attender of dental services						
Always	4.4 (5.6)	3.6 (4.4)	3.9 (5.2)	5 (10.0)	4 (8.0)	8 (16.0)
Sometimes	6.3 (6.5)	6.9 (7.0)	7.2 (7.3)	75 (16.1)	81 (17.4)	85 (18.3)
Never	10.3 (9.1)	10.4 (9.4)	11.0 (9.8)	116 (35.3)	106 (32.2)	111 (33.7)
*P* value	<0.001	<0.001	<0.001	<0.001	<0.001	<0.001

**Table 2:** Change in oral health impact profile (OHIP-14) score by socio-demographic characteristics and experience of oral conditions.

	Change in OHIP-14 Scores between Age 32 and 45			
	Worsened N Participants (%)	Stable no Change N Participants (%)	Improved N Participants (%)	*P* Value
Total	184 (21.8)	305 (36.1)	355 (42.1)	
Proportion of adulthood in low socioeconomic group				
<50%	138 (20.3)	250 (36.8)	292 (42.9)	
≥50%	46 (28.1)	55 (33.5)	63 (38.4)	0.090
Proportion of adulthood as routine attender of dental services				
Always routine	6 (12.0)	21 (42.0)	23 (46.0)	
Sometimes routine	94 (20.2)	190 (40.9)	181 (38.9)	
Never routine	84 (25.5)	94 (28.6)	151 (45.9)	0.001
	Mean change (sd)	Mean change (sd)	Mean change (sd)	
Number of decayed surfaces from 32 to 45 y	0.11 (0.45)	−0.40 (0.17)	−1.40 (0.30)	0.005
Number of filled surfaces from 32 to 45 y	0.54 (0.52)	2.29 (0.30)	1.72 (0.30)	0.109
Number of missing teeth from 32 to 45 y	2.06 (0.36)	0.83 (0.13)	1.53 (0.23)	0.017
Percent of sites with periodontal attachment loss 5 + mm from 32 to 45 y	4.12 (0.91)	3.20 (0.54)	2.42 (0.40)	0.274

**Table 3:** Change in impacts on oral health-related quality of life by participant socio-demographic characteristics and experience of oral conditions.

	Change in Prevalence of Impacts from Age 32 to 45				
	Resolved N (%)	Stable no Impacts N (%)	Incident Impacts N (%)	Stable Impacts N (%)	*P* Value
Total	101 (13.2)	529 (62.7)	119 (14.1)	85 (10.1)	
Proportion of adulthood in low socioeconomic group					
<50%	80 (11.8)	466 (68.5)	88 (12.9)	46 (6.8)	
≥50%	31 (18.9)	63 (38.4)	31 (18.9)	39 (23.8)	<0.001
Proportion of adulthood routine attender of dental services					
Always	5 (10.0)	37 (74.0)	8 (16.0)	0 (0)	
Sometimes	48 (10.3)	332 (71.4)	58 (12.5)	27 (5.8)	
Never	58 (17.6)	160 (48.6)	53 (16.1)	58 (17.6)	<0.001
	Mean change (sd)	Mean change (sd)	Mean change (sd)	Mean change (sd)	
Number of decayed surfaces from 32 to 45 y	−0.9 (0.5)	−0.7 (0.1)	0.3 (0.4)	−2.2 (1.2)	0.254
Number of filled surfaces from 32 to 45 y	1.7 (0.6)	2.2 (0.2)	1.1 (0.6)	−0.6 (0.8)	0.001
Number of missing teeth from 32 to 45 y	1.6 (0.4)	0.8 (0.1)	1.7 (0.4)	4.1 (0.7)	<0.001
Percent of sites with periodontal attachment loss 5 + mm from 32 to 45 y	3.8 (0.8)	2.0 (0.3)	2.2 (0.4)	10.6 (2.4)	<0.001

**Table 4:** Generalised estimating equation model for oral health impact profile (OHIP-14) scores from age 32 to 45 years.

	IRR for OHIP-14 Score (95% CI)		
	Unadjusted	Adjusted Stage I ^a^	Adjusted Stage II ^a^
Sex			
Male	-	-	-
Female	1.00 (0.90–1.12)	1.04 (0.96–1.16)	1.04 (0.94–1.16)
Childhood socioeconomic status			
High	-	-	-
Medium	1.31 (1.11–1.55)	1.19 (1.01–1.40)	1.16 (1.00–1.34)
Low	1.70 (1.40–2.06)	1.34 (1.11–1.62)	1.26 (1.05–1.50)
Proportion of adulthood in the lowest socioeconomic group			
<50%	-	-	-
≥50%	1.71 (1.52–1.92)	1.39 (1.22–1.58)	1.16 (1.03–1.31)
Proportion of adulthood as routine attender of dental services			
Never	-		-
Sometimes	0.64 (0.58–0.72)		0.85 (0.76–0.94)
Always	0.38 (0.28–0.50)		0.59 (0.44–0.80)
Number of decayed tooth surfaces	1.38 (1.28–1.48)		1.25 (1.16–1.35)
Number of filled tooth surfaces	0.98 (0.93–1.03)		1.08 (1.04–1.13)
Number of missing teeth	1.45 (1.29–1.64)		1.47 (1.29–1.68)
Percent of sites with periodontal attachment loss 5 + mm	1.10 (107–1.14)		1.03 (0.99–1.06)
Dry mouth			
No	-		-
Yes	1.34 (1.18–1.51)		1.30 (1.16–1.45)
Tooth-brushing			
Once or less per day	-		-
More than once per day	1.28 (1.17–1.40)		1.14 (1.04–1.25)

^a^ Controls for age and personality traits.

**Table 5:** Generalised estimating equation model for oral health impact profile (OHIP-14) impact prevalence from age 32 to 45 years.

	OR for OHIP-14 Impacts (95% CI)		
	Unadjusted	Adjusted Stage I ^a^	Adjusted Stage II ^a^
Sex			
Male	-	-	-
Female	1.11 (0.88–1.40)	1.34 (1.05–1.73)	1.37 (1.05–1.78)
Childhood socioeconomic status			
High	-	-	-
Medium	1.79 (1.26–2.56)	1.49 (1.04–2.11)	1.29 (0.92–2.37)
Low	3.05 (2.04–4.55)	1.97 (1.32–2.96)	1.59 (1.07–2.37)
Proportion of adulthood in the lowest socioeconomic group			
<50%	-	-	-
≥50%	2.96 (2.27–3.85)	1.98 (1.47–2.69)	1.29 (1.06–1.90)
Proportion of adulthood as routine attender of dental services			
Never	-		-
Sometimes	0.41 (0.32–0.52)		0.70 (0.54–0.92)
Always	0.25 (0.15–0.43)		0.59 (0.32–1.12)
Number of decayed tooth surfaces	2.53 (1.95–3.27)		1.64 (1.26–2.15)
Number of filled tooth surfaces	1.00 (0.90–1.11)		1.15 (1.03–1.27)
Number of missing teeth	2.76 (1.81–4.21)		2.76 (1.69–4.51)
Percent of sites with periodontal attachment loss 5 + mm	1.28 (1.15–1.43)		1.04 (0.91–1.20)
Dry mouth			
No	-		-
Yes	2.36 (1.77–3.14)		2.32 (1.66–3.25)
Tooth-brushing			
Once or less per day	-		-
More than once per day	0.56 (0.46–0.69)		0.75 (0.59–0.95)

^a^ Controls for age and personality traits.

## Data Availability

The Dunedin Study data are available on request to the Study Director by qualified scientists. Requests require a concept paper describing the purpose of data access, ethical approval at the applicant’s institution, and provision for secure data access. We offer secure access on the Duke University, Otago University, and King’s College London campuses.
